# Dynamics of Cultural Transmission in Native Americans of the High Great Plains

**DOI:** 10.1371/journal.pone.0112244

**Published:** 2014-11-05

**Authors:** Stephen J. Lycett

**Affiliations:** Department of Anthropology, University at Buffalo, The State University of New York, Amherst, New York, United States of America; Universidade do Algarve, Portugal

## Abstract

Culture is a phenomenon shared by all humans. Attempts to understand how dynamic factors affect the origin and distribution of cultural elements are, therefore, of interest to all humanity. As case studies go, understanding the distribution of cultural elements in Native American communities during the historical period of the Great Plains would seem a most challenging one. Famously, there is a mixture of powerful internal and external factors, creating-for a relatively brief period in time-a seemingly distinctive set of shared elements from a linguistically diverse set of peoples. This is known across the world as the “Great Plains culture.” Here, quantitative analyses show how different processes operated on two sets of cultural traits among nine High Plains groups. Moccasin decorations exhibit a pattern consistent with geographically-mediated between-group interaction. However, group variations in the religious ceremony of the Sun Dance also reveal evidence of purifying cultural selection associated with historical biases, dividing down ancient linguistic lines. The latter shows that while the conglomeration of “Plains culture” may have been a product of merging new ideas with old, combined with cultural interchange between groups, the details of what was accepted, rejected or elaborated in each case reflected preexisting ideological biases. Although culture may sometimes be a “melting pot,” the analyses show that even in highly fluid situations, cultural mosaics may be indirectly shaped by historical factors that are not always obvious.

## Introduction

Understanding how different factors influence the representation and organization of cultural (i.e., socially-learned) behaviors remains one of the central concerns of anthropology and the social sciences [1]−[5]. Influences on the representation of within- and between-group cultural practices are often suggested to include parameters such as geography, language, and ideology, among many others ([6]−[10]). However, testing the potency of such factors in specific cases remains imperative.

**Figure 1:**
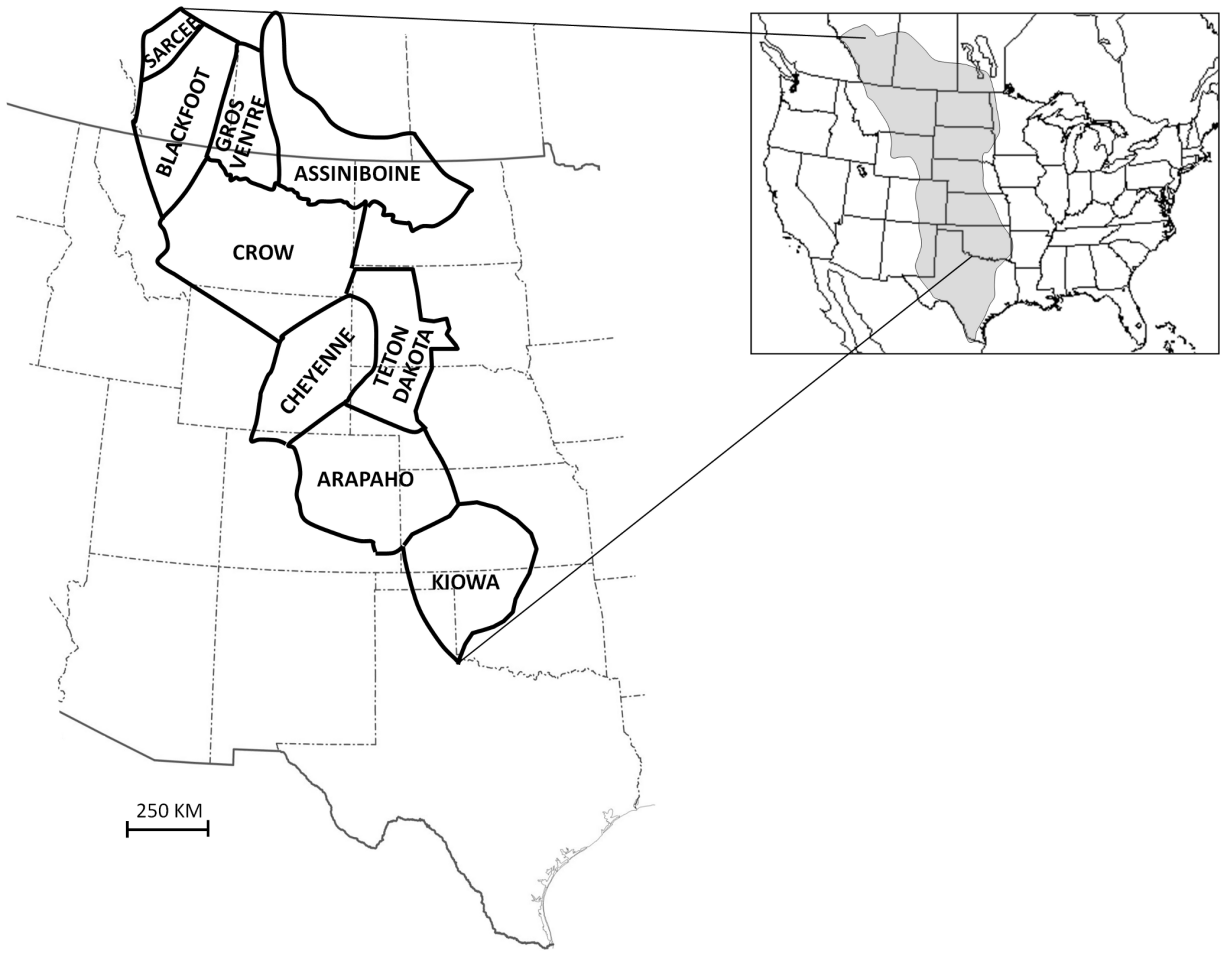
Schematic geographic distributions (after DeMallie (2001)) of the tribal groups considered here, *circa* mid-late 19th century. Shaded area on inset shows extent of Great Plains (both short-grass high plains and tall-grass prairie regions combined). Note: The Gros Ventre are also referred to as the Atsina.

As case studies go, attempting to understand the factors determining the distribution of cultural variations among Native American groups of the High Great Plains during the late 19th century would seem to present particular challenges. The High Plains is a semi-arid, grassland region which, until their decimation by European migrants, was home to large herds of migratory buffalo (*Bison bison*). By the 1700 s, the introduction of horses to the region had created new opportunities for more extensive pursuit of buffalo in the High Plains, an area running west from approximately the 100th meridian to the Rocky Mountains, and from southern Canada in the north, to west Texas in the south [11]−[13] (Figure 1). Famously, these factors created what became known as the “Great Plains culture” [12], [14]−[16]. Although the subregion of the Great Plains referred to as the “High Plains” is by no means environmentally uniform [11], [13], the region is united by a common set of ecological conditions that drew herds of buffalo and, ultimately, increased numbers of equestrian hunters [17]. Groups who came to occupy the High Plains adopted the nomadic pursuit of buffalo to the greatest extent, and exhibited traits that unite them as a particularized cultural phenomenon during the 19th century. Most notably, Wissler [14] based this on their mobility via use of horses and dog travois, buffalo hunting economy, absence of agriculture, use of tipis, lack of pottery production, adoption of Sun Dance, and elaborated working of rawhide and skins. The latter was particularly exemplified in geometric art including painting, beadwork and quillwork [16]. While none of these traits is unique to the High Plains, in combination they mark a particular historico-geographic phenomenon. This seeming cultural “homogeneity” was, however, the product of groups of people from at least six different language families, most of whom are known to have been recent migrants to the region [18], [19]. Such migratory episodes were also connected to events precipitated by European migrants in the east [20]. In effect, as a result of the newly created dynamics, the region became a “melting pot” for traditions, casting new cultural patterns over the High Plains.

In recent years it has been more greatly appreciated that cultural transmission theory, when united with statistical analyses of empirical data, provides an objective means of tackling seemingly intractable questions relating to the spread and diversification of cultural ideas ([2], [4], [10], [21]−[29]). Here, this framework is used to organize a series of quantitative analyses on data from the High Plains. Two sets of cultural trait variants are examined: moccasin decorations and attributes of the Sun Dance ceremony. Common data were available for nine tribes that occupied the High Plains during the mid-late 19th century (Figure 1). These tribes are among those who most extensively adopted equestrian nomadism, with an economy based around pursuit of buffalo.

Held over several days, the “Sun Dance” was a religious ceremony that took place annually in either the late spring or early summer [16], [30]. Its timing was broadly coincident with the onset of the summer hunting season and, in particular, the gathering together of the various bands of the tribe who had been dispersed during the fall and winter months. This ceremony spread across the Plains from tribe-to-tribe as part of the development of the cultural practices that came to characterize the region during the 1800 s [30], [31]. The ceremony itself was generally divided into distinct phases, the details of which would vary in given cases. As an affair involving the entire tribe, it is considered to have played a role in affirming group values and ideals ([31]: 165, [32]). Several authors have described its general form [16], [30], [31], typically noting the following features. The ceremony is initiated by an individual member of the tribe and preliminary ceremonies are undertaken. This can include preparation of regalia and other items to be used in the ceremony, and the rehearsal of songs. A forked tree used as a “center pole” is sought and felled, around which a structure is constructed. During this process the center pole is potentially the subject of specific treatment (e.g., treated as if it were an “enemy” felled in battle). A bundle of brushwood along with effigies or other ceremonial items is placed in the fork of a branch at the top of the center pole prior to it being raised in position. Thereafter, an altar may be built within the structure that is constructed. Several days of dancing and singing are undertaken, along with the observance and performance of other customs and ceremonies. The “self-torture” element via skin piercing that made the ceremony globally famous was given particular emphasis in only certain groups (most notably the Teton Dakota), although self-sacrifice in the form of thirsting and fasting were common elements [30], [31]. Despite this general form, the details and specifics of all these features vary in different tribes. The generic name “Sun Dance” also conceals the fact that names for the ceremony varied in different tribes [33]. Indeed, it has been also noted that its outward or objective features are more consistent across tribes of the Plains than those associated with its meaning or ideological elements [16], [30], [31]. Data describing variation in a total of 82 traits for this ceremony [30] were used for the analyses.

**Figure 2:**
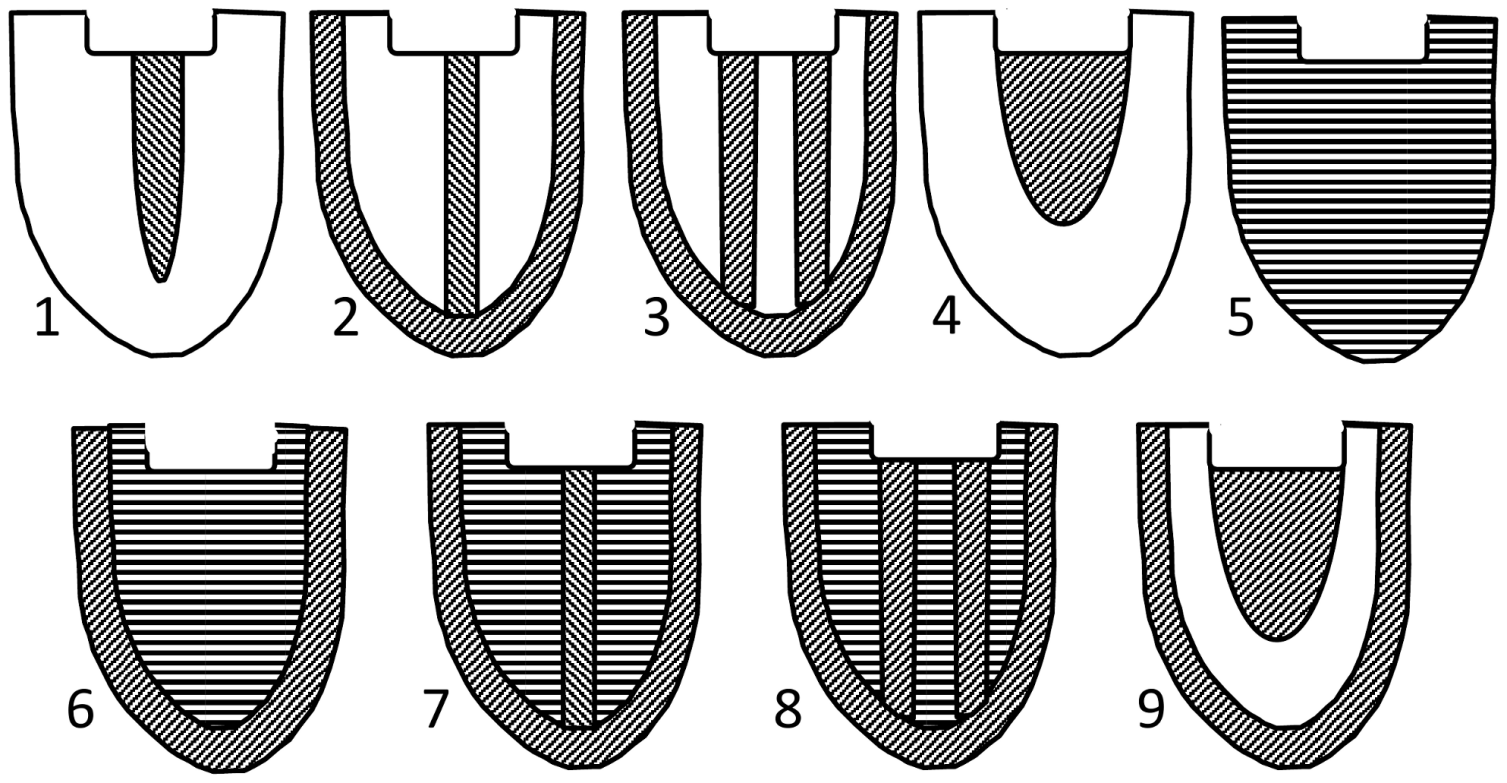
Examples of moccasin decoration types. 1) central bar without border; 2) border and central bar extending to toe; 3) border and two centered parallel lines; 4) a central U-shaped or other decorative figure on top portion of upper; 5) covered upper without border; 6) border and central area; 7) same as 6 but with central bar; 8) border and central area with two parallel lines; 9) border with central design at top of upper (Redrawn and modified after Wissler 1927).

The second set of cultural attributes examined were variations in the geometric/positional arrangement of bead and quillwork decorations on moccasins (Figure 2). Beads were of European manufacture, again indicative of the role of both internal and external factors in constituting certain cultural characteristics of the region [34]. The production of such decorations was undertaken by women, the details of which were learned from other women [35], [36], [37]. Data for moccasin decorations were taken from ref. [38].

Here, the data from these two sets of cultural variants were used to compile similarity/dissimilarity patterns among the nine tribal groups considered. Thereafter, a series of statistical analyses were undertaken to determine how particular dynamics of transmission might have influenced the representation of traits in different tribes on the High Plains. Of particular interest here are factors that conform to selectively neutral and selectively biased pathways of transmission between different tribes. Cultural distances between each of the nine tribes considered can be examined for causal factors of similarity and dissimilarity by comparing statistically (using Mantel tests) the cultural datasets against “model matrices” representing hypothesized determinants [9].

As has long been recognized [39], one of the primary potential determinants of cultural similarity between communities is their geographic proximity. This is because-all else equal-how close people are in geographic space can be expected to determine their likelihood and intensity of interaction. Several studies have shown the strong effect that geographic distance can have on cultural similarity, sometimes even cross-cutting known linguistic divides [7], [9], [27]. Geographic spacing therefore provides a primary hypothesis for predicting the cultural distance between different tribes, whereupon cultural trait representation is simply a function of mutation (innovation and/or transmission error), drift (stochastic traits loss), and migration (between-group interactions). Such a relationship between geography and cultural similarity is to be expected provided that selective factors are not biasing the representation of traits. This is directly analogous to the manner in which “isolation-by-distance” provides an explanatory model for neutral genetic similarity and dissimilarity between related populations [40], [41].

In the case of the Great Plains, however, linguistic diversity must also be considered. Much of this diversity, of course, was caused by the influx of people during the post-contact era. Even the subset of nine tribes considered here, represent groups whose languages belong to four distinct language families [19]. Hence, data for the moccasin decorations and the Sun Dance were also compared to model matrices representing linguistic similarity between groups. Linguistic relatedness of different groups is also likely to reflect other factors of historical relatedness, most notably genetics [40]. Hence, when language does not correlate with geography, a correlation between linguistic patterning and cultural variation may be a proxy for other factors influencing the distribution of cultural variants, such as the operation of ideological or religious biases shared by groups that were once closely related enough to be linguistically cognate. The comparison of a cultural dataset representing clothing decoration versus one associated with a religious ceremony may be particularly informative in this regard.

## Results

Mantel tests indicated no significant relationship between matrices describing Jaccard distances between tribes when moccasin variants were compared directly with the Sun Dance data (*r* = 0.326; *p* = 0.202). This immediately indicates that the two sets of traits do not vary in the same manner across the different tribes, suggesting that each of these cultural phenomena have been subject to different historical processes affecting their distribution.

Table 1 shows that moccasin decorations show a significant fit to geographic distances between tribes, using two different measures of geographic proximity (see Methods). However, these data are not significantly correlated with linguistic distances between tribes. This would indicate that patterns of variation in moccasin decorations fit a model of geographic patterning.

**Table 1:** Mantel test results comparing cultural datasets to the model matrices for geography and language (significant values in bold).

	Geographic distances	Border share	Language M1	Language M2	Language M3
	*r*/*p*	*r*/*p*	*r*/*p*	*r*/*p*	*r*/*p*
Moccasins	−0.444/**0.020**	−0.333/**0.021**	0.160/0.190	0.155/0.192	0.140/0.208
Sun Dance	−0.424/**0.005**	−0.469/**0.002**	0.506/**0.002**	0.482/**0.004**	0.415/**0.013**

The Sun Dance data also exhibit a significant correlation with geography (Table 1). However, these data also exhibit a significant relationship with the linguistic data, using three variants of coding linguistic distances. It is also noteworthy that linguistic affinities and geographic distances are not correlated (*r*_LanguageMatrix1_ = −0.115, *p*_LanguageMatrix1_ = 0.239; *r*_LanguageMatrix2_ = −0.104, *p*_LanguageMatrix2_ = 0.263; *r*_LanguageMatrix3_ = −0.079, *p*_LanguageMatrix3_ = 0.319). Moreover, Sun Dance variants correlate with all three languages matrices, even when controlling for geographic distance via partial Mantel tests (*r*_Matrix1Partial_ = 0.508, *p*_Matrix1Partial_ = 0.002; *r*_Matrix2Partial_ = 0.486, *p*_Matrix2Partial_ = 0.003; *r*_Matrix3Partial_ = 0.423, *p*_Matrix3Partial_ = 0.004). These analyses demonstrate that patterns in the Sun Dance data exhibit a robust relationship with linguistic distances between tribes, even when geography is controlled for.

In sum, the Mantel tests indicate that the moccasin data exhibit a fit to the geographic pattern, as expected under a model of trait patterning mediated primarily by spatial interrelationships between different tribes. Analyses of the Sun Dance data, however, indicate that cultural patterns exhibited in the different tribes are not merely the product of geographic relationships between them. Rather, trait distribution also appears to have been affected by some other factor; one which covaries with the (more ancient) linguistic affinities of each tribe.

Given that the moccasin data are primarily explained by geography whereas the Sun Dance data also strongly fit a model of linguistic distances, the possibility of selection biases in the Sun Dance data needs to be further considered. This is especially the case given that the Sun Dance data are strongly correlated with linguistic data, even when geography is controlled for. Moreover, because most 19th century High Plains tribes were recent migrants to the region [12], [19], their geographic locations during that time period cannot reflect ancient influences on cultural factors; however, other historical factors reflected in the linguistic affinities of the various tribes may do, and thus explain the empirical pattern observed. It has been suggested that when a cultural trait has been subject to selection it may exhibit a significantly greater or lower level of overall between-group diversity compared to one that has been subject to more selectively neutral factors [42]. This is because negative (or “purifying”) selection may result in significantly lower between-group diversity compared with a trait subject to more neutral influences, whereas a trait that has been subject to positive (or “diversifying”) selection biases can be expected to exhibit significantly greater between-group diversity compared to the more neutral pattern. Strictly, this test alone cannot be considered evidence of selection, since other factors may also contribute to a discrepancy in levels of diversity in two sets of comparable traits [43], [44]. However, in the face of the Mantel test results, which indicate that Sun Dance variants correlate with the linguistic affinities of the various tribes (yet language and geography are not correlated with each other), the possibility that linguistic affiliations are acting as a proxy for ancient, historical and culturally-inherited biases which, in turn, are having a selective influence on the distribution of these traits needs to be further considered. It is this manner of post-hoc test-in light of the Mantel test results–that this second set of analyses is being used here.

Intertribe distances (Jaccard measures) for moccasin and Sun Dance variants were compared using three different nonparametric statistical tests: a Sign test for paired observations, a permutated Wilcoxon test for paired observations, and a permutated Mann-Whitney U-test (see Methods). All three tests indicated statistically significant differences between the intertribe distances observed in the Sun Dance data compared to those seen in the moccasin data (Table 2). Moreover, given that measures of central tendency for the Sun Dance data (mean = 0.317; median = 0.307) were lower than those observed for the moccasin data (mean = 0.513; median = 0.555), this would indicate that the Sun Dance traits were subject to purifying (i.e., negative) selection across the various tribes.

**Table 2:** Results of nonparametric tests comparing moccasin distances against Sun Dance distances.

Test	Test statistic	Probability
Wilcoxon signed ranks	*W* = 575	Monte Carlo *p*<0.0001
Sign	*r* = 25	*p* = 0.029
Mann-Whitney	*U* = 370	Monte Carlo *p* = 0.0018

## Discussion

“Culture” is the conglomeration of information, knowledge, ideas, and beliefs, shared by communities and transmitted by social interaction [4], [40], [45]. This shared property characterizes all human societies. Communities that came to occupy the High Great Plains during the 19th century exemplify the manner in which humans can take existing ideas, elaborate them, combine them with new ones, pass them successfully between groups and create novel, distinct patterns, visible over temporal and spatial scales. Attempting to examine the role of specific factors in creating cultural patterning under such historically-contingent, transient, and dynamic conditions, however, presents a challenge.

Here, analyses have shown the presence of distinct processes operating and ultimately influencing the representation of cultural traits in different tribes. Patterns of similarity and difference in moccasin designs among different tribes show a statistically significant relationship with the model of geographic relationships between tribes. No statistical effect of language affiliation on the distribution of moccasin decorations was detected. This indicates that the representation of moccasin decoration types among groups is, in this case, most strongly determined by whether or not that trait is present in another, geographically proximate, tribe. In other words, selection biases have not disrupted the distribution of these decorations to the extent that they deviate significantly from a pattern predictable on the basis of the geographic relationships between tribes alone. These results would seem to reaffirm the role of intergroup relations in creating a pattern of shared cultural similarity over the region during the 19th century, which many have previously discussed [14], [16], [18], [20].

Behavioral variation between tribes in terms of their practice of the religious ceremony of the Sun Dance also indicated a statistical relationship with geography, again reiterating the role of intertribe transmission in creating the phenomenon historically labelled as the “Great Plains culture” [14], [16]. However, patterns of intertribe variation in Sun Dance elements also exhibited a statistically significant relationship with linguistic affinities between different tribes. Ordinarily, this pattern might simply be attributed to the fact that tribes with more mutually comprehensible languages were able to more effectively transmit the behavioral variants among themselves. Here, however, language patterns were found not to correlate with geographic patterns and, moreover, the statistical relationship between Sun Dance patterning and linguistic affiliation was found to still hold even when geography was controlled for. The relationship between Sun Dance variations and language patterns is, therefore, in this instance puzzling.

The statistical results obtained for the Sun Dance data are, however, explicable in terms of the operation of culturally inherited selective biases, for which linguistic affiliations are providing an observable proxy. This is further supported in the second set of analyses, which indicate that intertribe distances are significantly lower than those exhibited in the moccasin data; a pattern which is consistent with the operation of negative (or “purifying”) selection biases on the Sun Dance variants. That is, although the results again suggest a role for geography in mediating intergroup transmission, details of Sun Dance variants observed in each tribe–the details of what was accepted, rejected or elaborated in individual tribes-also reflected preexisting ideological biases. The pattern-match with language in this case would, therefore, reflect not a role for language directly, but the presence of ancient ideological biases, which ultimately exert an influence on the distribution of variants seen in different tribes as the Sun Dance spread across the High Plains.

Previous scholars have noted that the observable features of the Sun Dance were more easily recorded than its meanings and ideological components across tribes [30], [31]. Yet here we see evidence for the role of (historical) culturally-inherited ideological features acting as biases, and so shaping the form of the Sun Dance in different tribes as it spread across the High Plains. Anthropologists have for a long time suggested that religious practices affirm social ties and values [46]. However, equally-as with languages-religious practices rely on mutual cooperation; meaning in either is corrupted by change, and conservatism in the transmission of religious beliefs is documented [47], [48]. The results here indicate that conservatism in transmission of ideological beliefs, down the same historical lines as those seen in linguistic affiliations, shaped at least some of the patterns that created cultural elements observed among different tribes of the Great Plains.

Most commentators on transmission of Sun Dance practices have previously emphasized the role of geographic proximity, drawing attention to the idea that groups closer together tend to share more traits, those further apart, less [30], [49], [50]. The analyses undertaken here again confirm the role of geographically-mediated intertribe transmission in facilitating the spread of the Sun Dance. Under these conditions, however, we might reasonably have expected religious differences between groups simply to create considerable between-group diversity (i.e., through diversifying selection) in terms of the details of this ceremony (see e.g., [4]: 219). Indeed, Spier [30] in his original discussion of the Sun Dance, speculated on reasons as to why certain traits might have been accepted, rejected, or elaborated in the various cases, including accordance with existing cultural attitudes. However, he was forced to conclude that as a result “of leveling produced by long continued cross transmission … there is no indication contained in the character of the traits why a particular object is copied and another rejected” ([30]: 500). Here, however, the analyses identify the role of deeply-rooted ideological biases, historically inherited down generations in accordance with the linguistic affiliations of the different groups involved.

The role of historical, culturally inherited biases in leading to statistically identifiable patterns in the geographic distribution of religious practices is probably an area ripe for future research. To allude to but one example, consider the Christian festival of “Christmas,” which has many common recognizable elements wherever it is observed across the globe, yet has subtleties of form and practice across the various communities and countries in which it is observed [51], [52]. In light of the results reported here, it is reasonable to ask to what extent these patterns are shaped by preexisting and culturally inherited ideological factors as opposed to merely intercommunity contact. Equally, as globalization increases, new cultural patterns will inevitably spread over existing, culturally antecedent, sets of conditions. The results here suggest that however pervasive such ideas may seem, where new ideas come into contact with older ones, longstanding ideas within communities may yet influence the shape that these new patterns take.

## Conclusions

The cultural patterns created by Native Americans on the Great Plains during the historical period might appear to exemplify the idea that cultural processes can be a “melting pot” for existing traditions creating a level of uniformity via intergroup transmission. However, monocausal explanations-be they language, historical precedence, intergroup migration, or ecology-for the distribution of individual elements within wider cultural mosaics are unlikely to prove fully satisfactory. Here, we see evidence for two different types of dynamics operating to shape the form of a particular cultural mosaic operating in one subregion of the Great Plains during the late 19th century. One of these dynamics is geographically-mediated transmission pathways, the other is the existence of more ancient, culturally-inherited ideological biases that act to shape the mosaic in ways that cannot be explained solely on the basis of geography alone. These findings remind us of the need to avoid overly simplistic scenarios when attempting to understand cultural entities in all of their forms.

## Materials and Methods

### Tribes examined

A total of nine tribal groups were examined here for which common data for both moccasin decorations and Sun Dance attributes were available (Figure 1). The tribes examined account for nine (out of 11) of Wissler’s [14] “typical” Plains tribes (those missing are the Comanche and Plains Apache of the Southern Plains). The taxonomic designation of a “Plains culture” or indeed a “typical” group of tribes (*sensu* ref. [14]) is, of course, an artificial phenomenon, oversimplifying relations between groups within and outside of the Great Plains region [20], [53]. However, the neighboring groups examined here are among those that most extensively adopted equestrian nomadism based around a buffalo hunting economy and this, in combination with commonality of available Sun Dance and moccasin data for all nine groups, keeps the analyses within prescribed geographic limits. For the purposes of analysis, northern and southern bands of the Cheyenne were treated as a single group. All data utilized in the analyses are publicly available in the references described below.

### Sun Dance data

Data were taken from Spier’s [30] comparative study of Plains tribes. Spier’s report [30] is widely regarded as the definitive study of the Sun Dance ([31]: 165). There have been previous quantitative analyses of Spier’s [30] data, including speculations on geographic patterning or lack thereof [49], [50], but these have never explicitly included spatial data, nor been formally compared with another cultural dataset. Data are in the form of presence/absence for each particular ceremonial variant. For the purposes of analysis, data were converted into a distance matrix describing patterns of similarity/dissimilarity between all pairs of tribes (Table S1). Here, Jaccard measures were used to describe the distances, since this measure is suitable for presence/absence data, and in focusing on differences in shared presences rather than absences, helps control for absences that may simply be the result of observational bias [9], [54]. Similarly, this is also the most appropriate measure when comparing the relative extent of diversity in two sets of trait categories [42]. A small number of tribal observations (8 out of 738, or 1%) were originally coded as questionable (?) by Spier [30]. Conservatively, this small number of data points were treated by pairwise deletion in computing the Jaccard distances; whereupon if a data point was coded as questionable for one the variables in a pair, that variable was excluded from the calculation of the total distance between those groups. However, performing all analyses with questionable (?) instances coded as present resulted in an identical statistical pattern.

### Moccasin data

Data were taken from Wissler’s [38] summary tables describing the presence of decoration types on hard-soled (two-piece) moccasins, as recorded in the collections of the American Museum of Natural History, New York (n = 387 examples for the nine tribes considered here). Wissler [38] notes that these decorations were produced while the uppers were flat, prior to attachment to the sole [35]. A Mantel test (see below) of a Euclidean distance matrix based on sample sizes for each group showed no significant relationship with distances (based on Jaccard measures) between tribes based on decoration types (*r* = −0.252; *p* = 0.175). This confirms Wissler’s [38] earlier assertion that decoration patterns represented for each tribe are not a function of sample size. Wissler [38] listed 10 decorative variants present among the tribes considered here. For purposes of analysis, presence/absence data were again transformed into a distance matrix describing patterns of similarity/dissimilarity between all pairs of tribes using the Jaccard distance measure (Table S2).

### Calculation of geographic relationships between tribes

The geographic position and extent of tribal ranges is taken from ref. [18], who notes that the map is a schematic approximation of relative positions. In reality the situation was fluid in terms of movement, and tribal boundaries shifted somewhat over time. Hence, the base map is merely a general statement of relative geographical relationships between groups *circa* the mid-late 19th century. However, when central points within these approximations are treated as point coordinates (as here) about which groups moved and redistributed themselves, they provide potential for spatial analysis. That is, center points of these approximations, at a scale of the entire region reflect the major aspects of spatial distances between these groups during the latter part of the 19th century. Taking the base map indicated, geographic coordinates at the center of each territory (Table S3) were used to compute a geographic distance matrix using the freely available program published by the American Museum of Natural History (http://biodiversityinformatics.amnh.org/open_source/gdmg/index.php). Following ref. [9], a second measure of geographic proximity was also computed based on whether tribes shared a territorial boundary or not, using the same base map as that for the geographic distances. This latter data matrix was coded in binary form where 1 indicated no sharing of a border while 0 indicated proximity via presence of a shared border.

### Linguistic affinities of tribes

It has been noted in regard to the tribes of the Great Plains that “linguistic diversity was much greater than the diversity of other aspects of their cultures and must be a retention from a time when they had less contact with each other and were culturally more distinct” ([19]: 61). Linguistic designations for the nine tribes were converted into a model matrix, quantitatively representing the relative linguistic similarity/dissimilarity of all groups. The method utilized broadly follows that of ref. [7] (for similar application see ref [9]). Language classification followed the consistently described patterns in refs. [19], [55]−[58]. These classifications were converted into a distance matrix (“Language Matrix 1”) using the following scheme: 5% for groups that spoke languages from entirely different families; 50% for groups from the same language family but belonging to different subgroups; 80% for groups speaking languages of the same subgroup within a family. Since this scheme creates a “model” of distance, two further matrices were concurrently tested alongside the primary matrix in order to ensure that results were robust to slight deviations. One of the additional matrices (“Language Matrix 2″) coded greater similarity (95%) in the Arapaho-Gros Ventre [Atsina] (Arapahoan) and the Assiniboine-Teton (Dakotan) subgroups within their respective language families than in the primary matrix. A third matrix (“Language Matrix 3”) coded a level of only 30% for languages of the same family, while keeping all other interfamily (5%) and subgroup (80%) codings consistent with the primary matrix. All three language matrices are shown in Tables S4–S6.

### Mantel Matrix tests

Using the data described, correlations between distance matrices were examined statistically. Since matrices violate the assumptions of traditional correlation statistics, all matrices were compared using Mantel tests [59]. Statistical significance in Mantel tests is determined via permutations of the original data (here, involving 10,000 randomizations) [60]. Partial Mantel tests are also possible, whereby the relationship between two matrices can be assessed while controlling for the effect of a third confounding factor [61]. All Mantel tests were undertaken using the freely available software PASSaGE 2 (http://www.passagesoftware.net/; [62]), where the critical alpha level was set at α = 0.05. When applied in a context of phylogenetic comparative analyses (i.e., where cultural data are expected to correlate with a known phylogenetic pattern as a null hypothesis), use of Mantel tests has drawn criticism (e.g., [63], [64]) due to suggestions that in such applications they may have low statistical power and associated error rates. In the case of direct phylogenetic comparisons these problems are most pertinent where geographical and historical (i.e., phylogenetic) distance matrices are highly correlated (see [64]: 201). Here, however, there is a demonstrable lack of autocorrelation between geography and the historical relatedness of the different groups, as indicated by linguistic affiliation (see Results), warranting the use of Mantel tests in this instance. Notably, given the lack of relationship between the geographic distribution of tribal groups and their linguistic affinities, no (linguistically concordant) tree-like structure regarding the geographic distribution of cultural variants need be assumed a priori.

### Testing for selection

A statistically significant difference (α = 0.05) between two sets of distances can indicate selection when one set of distances is attributable to a neutral pattern of variation [42]. As noted earlier, when distances are significantly greater than those fitting the neutral pattern, this would indicate positive (or “diversifying”) selection. Conversely, when distances are significantly lower than those fitting the neutral pattern, this would indicate negative (or “purifying”) selection. Following ref. [42], three sets of conservative nonparametric tests were undertaken. A Wilcoxon signed ranks test (with randomization) for paired observations (Monte Carlo with 99,999 randomizations), a Sign test for paired observations, and a Mann-Whitney U-test with randomization (Monte Carlo with 10,000 random assignments).

## Supporting Information

Table S1 **Sun Dance Jaccard distances.**
(DOCX)

Table S2 **Moccasin Jaccard distances.**
(DOCX)

Table S3 **Geographical coordinates of tribal groups.**
(DOCX)

Table S4 **Language Matrix 1.**
(DOCX)

Table S5 **Language Matrix 2.**
(DOCX)

Table S6 **Language Matrix 3.**
(DOCX)
