# Supplementary material for: Dynamics of Cultural Transmission in Native Americans of the High Great Plains
Source: PLoS One. 2014 Nov 5;9(11):e112244. doi: 10.1371/journal.pone.0112244 (PMC4221622; doi:10.1371/journal.pone.0112244)
Supplement: Table S1 — Sun Dance Jaccard distances. (DOCX) [file pone.0112244.s001.docx]

**Sun Dance Jaccard distances**

|  | Arapaho | Assiniboine | Gros_Ventre | Blackfoot | Cheyenne | Crow | Teton_Dakota | Kiowa | Sarcee |
| --- | --- | --- | --- | --- | --- | --- | --- | --- | --- |
| Arapaho | 1 | 0.29825 | 0.50847 | 0.40625 | 0.62903 | 0.31148 | 0.38235 | 0.38983 | 0.24138 |
| Assiniboine | 0.29825 | 1 | 0.31707 | 0.31707 | 0.21818 | 0.30556 | 0.25532 | 0.17073 | 0.26667 |
| Gros_Ventre | 0.50847 | 0.31707 | 1 | 0.47917 | 0.36667 | 0.36957 | 0.2931 | 0.26 | 0.2619 |
| Blackfoot | 0.40625 | 0.31707 | 0.47917 | 1 | 0.40678 | 0.31915 | 0.30508 | 0.18182 | 0.44737 |
| Cheyenne | 0.62903 | 0.21818 | 0.36667 | 0.40678 | 1 | 0.25862 | 0.35938 | 0.33929 | 0.32 |
| Crow | 0.31148 | 0.30556 | 0.36957 | 0.31915 | 0.25862 | 1 | 0.30769 | 0.23913 | 0.20513 |
| Teton_Dakota | 0.38235 | 0.25532 | 0.2931 | 0.30508 | 0.35938 | 0.30769 | 1 | 0.23636 | 0.20408 |
| Kiowa | 0.38983 | 0.17073 | 0.26 | 0.18182 | 0.33929 | 0.23913 | 0.23636 | 1 | 0.24324 |
| Sarcee | 0.24138 | 0.26667 | 0.2619 | 0.44737 | 0.32 | 0.20513 | 0.20408 | 0.24324 | 1 |
