# Supplementary material for: Dynamics of Cultural Transmission in Native Americans of the High Great Plains
Source: PLoS One. 2014 Nov 5;9(11):e112244. doi: 10.1371/journal.pone.0112244 (PMC4221622; doi:10.1371/journal.pone.0112244)
Supplement: Table S2 — Moccasin Jaccard distances. (DOCX) [file pone.0112244.s002.docx]

**Moccasin Jaccard distances**

|  | Arapaho | Assiniboine | Gros_Ventre | Blackfoot | Cheyenne | Crow | Teton_Dakota | Kiowa | Sarcee |
| --- | --- | --- | --- | --- | --- | --- | --- | --- | --- |
| Arapaho | 1 | 0.75 | 0.625 | 0.75 | 0.55556 | 0.8 | 0.77778 | 0.11111 | 0.625 |
| Assiniboine | 0.75 | 1 | 0.83333 | 1 | 0.33333 | 0.6 | 0.55556 | 0.14286 | 0.83333 |
| Gros_Ventre | 0.625 | 0.83333 | 1 | 0.83333 | 0.22222 | 0.5 | 0.44444 | 0.16667 | 0.66667 |
| Blackfoot | 0.75 | 1 | 0.83333 | 1 | 0.33333 | 0.6 | 0.55556 | 0.14286 | 0.83333 |
| Cheyenne | 0.55556 | 0.33333 | 0.22222 | 0.33333 | 1 | 0.6 | 0.55556 | 0.14286 | 0.375 |
| Crow | 0.8 | 0.6 | 0.5 | 0.6 | 0.6 | 1 | 0.8 | 0.2 | 0.5 |
| Teton_Dakota | 0.77778 | 0.55556 | 0.44444 | 0.55556 | 0.55556 | 0.8 | 1 | 0.11111 | 0.44444 |
| Kiowa | 0.11111 | 0.14286 | 0.16667 | 0.14286 | 0.14286 | 0.2 | 0.11111 | 1 | 0.16667 |
| Sarcee | 0.625 | 0.83333 | 0.66667 | 0.83333 | 0.375 | 0.5 | 0.44444 | 0.16667 | 1 |
