# Supplementary material for: Dynamics of Cultural Transmission in Native Americans of the High Great Plains
Source: PLoS One. 2014 Nov 5;9(11):e112244. doi: 10.1371/journal.pone.0112244 (PMC4221622; doi:10.1371/journal.pone.0112244)
Supplement: Table S3 — Geographical coordinates of tribal groups. (DOCX) [file pone.0112244.s003.docx]

|  | LATITUDE | LONGITUDE |
| --- | --- | --- |
| Arapaho | 39.9 | -103.1 |
| Assiniboine | 48.99 | -103.67 |
| Gros Ventre | 50.87 | -109.24 |
| Blackfoot | 51.09 | -113.77 |
| Cheyenne | 43.05 | -106.25 |
| Crow | 46.67 | -110.65 |
| Teton Dakota | 44 | -101.5 |
| Kiowa | 36.94 | -99.03 |
| Sarcee | 53.45 | -115.22 |
