# Supplementary material for: Dynamics of Cultural Transmission in Native Americans of the High Great Plains
Source: PLoS One. 2014 Nov 5;9(11):e112244. doi: 10.1371/journal.pone.0112244 (PMC4221622; doi:10.1371/journal.pone.0112244)
Supplement: Table S5 — Language Matrix 2. (DOCX) [file pone.0112244.s005.docx]

**Language Matrix 2**

|  | Arapaho | Assiniboine | Gros_Ventre | Blackfoot | Cheyenne | Crow | Teton_Dakota | Kiowa | Sarcee |
| --- | --- | --- | --- | --- | --- | --- | --- | --- | --- |
| Arapaho | 0 | 5 | 95 | 50 | 50 | 5 | 5 | 5 | 5 |
| Assiniboine | 5 | 0 | 5 | 5 | 5 | 50 | 95 | 5 | 5 |
| Gros_Ventre | 95 | 5 | 0 | 50 | 50 | 5 | 5 | 5 | 5 |
| Blackfoot | 50 | 5 | 50 | 0 | 50 | 5 | 5 | 5 | 5 |
| Cheyenne | 50 | 5 | 50 | 50 | 0 | 5 | 5 | 5 | 5 |
| Crow | 5 | 50 | 5 | 5 | 5 | 0 | 50 | 5 | 5 |
| Teton_Dakota | 5 | 95 | 5 | 5 | 5 | 50 | 0 | 5 | 5 |
| Kiowa | 5 | 5 | 5 | 5 | 5 | 5 | 5 | 0 | 5 |
| Sarcee | 5 | 5 | 5 | 5 | 5 | 5 | 5 | 5 | 0 |
